# Supplementary material for: The ultra-thin, minimally invasive surface electrode array NeuroWeb for probing neural activity
Source: Nat Commun. 2023 Nov 4;14:7088. doi: 10.1038/s41467-023-42860-9 (PMC10625630; doi:10.1038/s41467-023-42860-9)
Supplement: Supplementary file 1 — Supplementary Information [file 41467_2023_42860_MOESM1_ESM.pdf]

Supplementary Information

The ultra-thin, minimally invasive surface electrode array NeuroWeb for probing neural activity

Jung Min Lee[1]†, Young-Woo Pyo[1,2]†, Yeon Jun Kim[2], Jin Hee Hong[2,3], Yonghyeon Jo[2,3], Wonshik Choi[2,3], Dingchang Lin[4], and Hong-Gyu Park[1]*

[1]Department of Physics and Astronomy, and Institute of Applied Physics, Seoul National University, Seoul 08826, Republic of Korea.

[2]Department of Physics, Korea University, Seoul 02841, Republic of Korea.

[3]Center for Molecular Spectroscopy and Dynamics, Institute for Basic Science, Seoul 02841, Republic of Korea.

[4]Department of Materials Science and Engineering, Johns Hopkins University, Baltimore, Maryland 21218, USA.

*Correspondence to: hgpark@snu.ac.kr

†These authors contributed equally to this work.

This file includes:

Supplementary Figures 1 to 26

Supplementary Table 1

Supplementary References

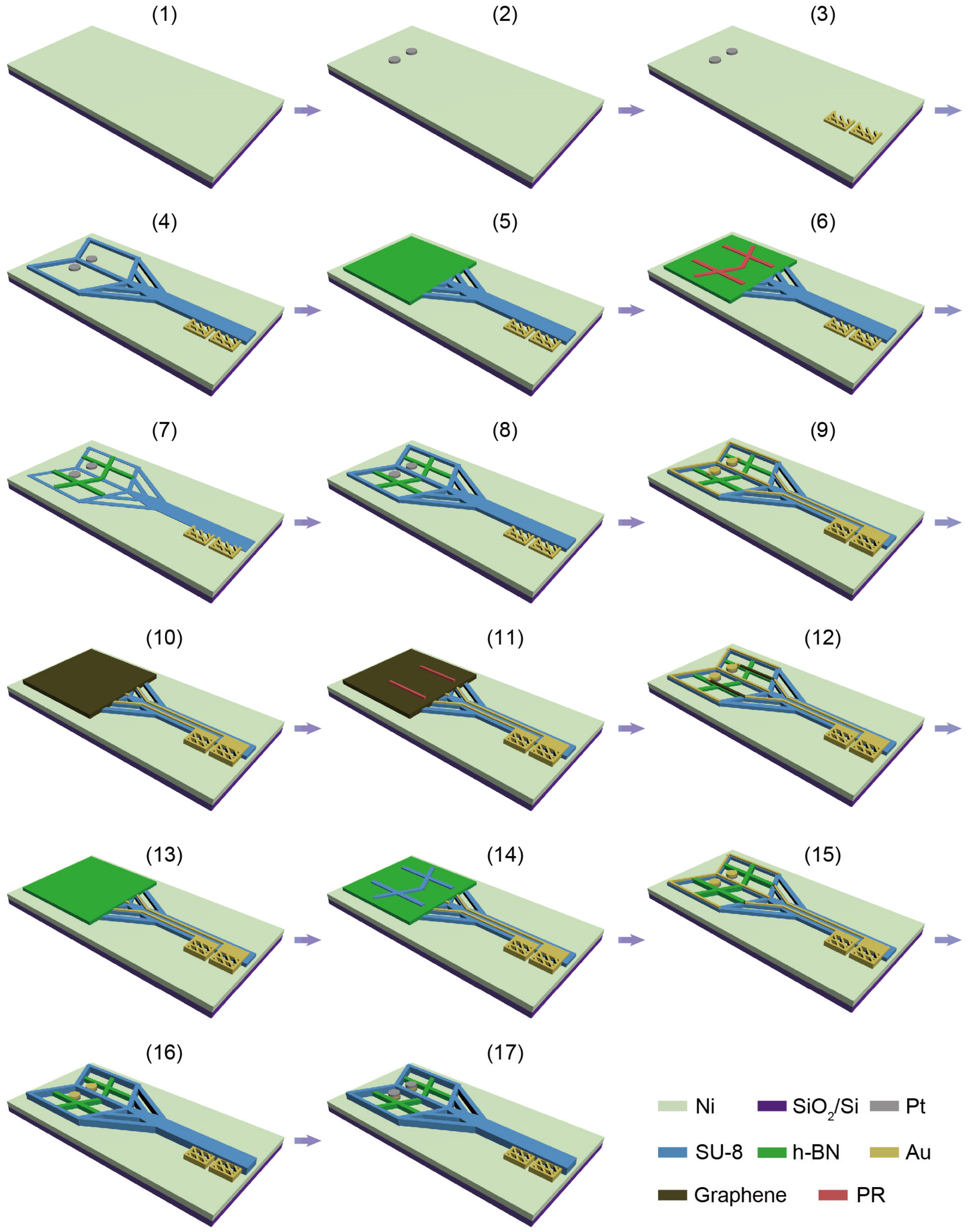

Supplementary Fig. 1. Fabrication procedure. NeuroWeb was fabricated using the following fabrication steps: (1) deposition of a 100 nm-thick Ni sacrificial layer on a SiO_2/Si substrate; (2)

fabrication of 50 nm-thick bottom Pt electrodes on a Ni sacrificial layer; (3) fabrication of 100 nm-thick Au I/O pads on a Ni sacrificial layer; (4) patterning of the SU-8 layer to find the position of the active region of NeuroWeb; (5) transferring multilayer h-BN to the active region of NeuroWeb using the PMMA wet transfer method; (6) patterning of the PR etch mask; (7) fabrication of the bottom h-BN passivation layer for Gr interconnects using O_2 plasma etching; (8) fabrication of the SU-8 layer acting as the bottom passivation layer for metal interconnects and as the supporting backbone for the supporting region; (9) deposition of Au interconnects for connecting Gr and I/O pads, and for use in the supporting region; (10) transferring three layers of Gr to the bottom h-BN using the PMMA wet transfer method; (11) patterning of the PR etch mask; (12) fabrication of Gr interconnects using O_2 plasma etching; (13) transferring multilayer h-BN to the active region using the PMMA wet transfer method; (14) patterning of the SU-8 etch mask; (15) fabrication of the top h-BN passivation layer for Gr interconnects using O_2 plasma etching; (16) fabrication of the SU-8 layer acting as the top passivation layer for metal interconnects; (17) fabrication of the top Pt electrodes to form double-sided recording electrodes.

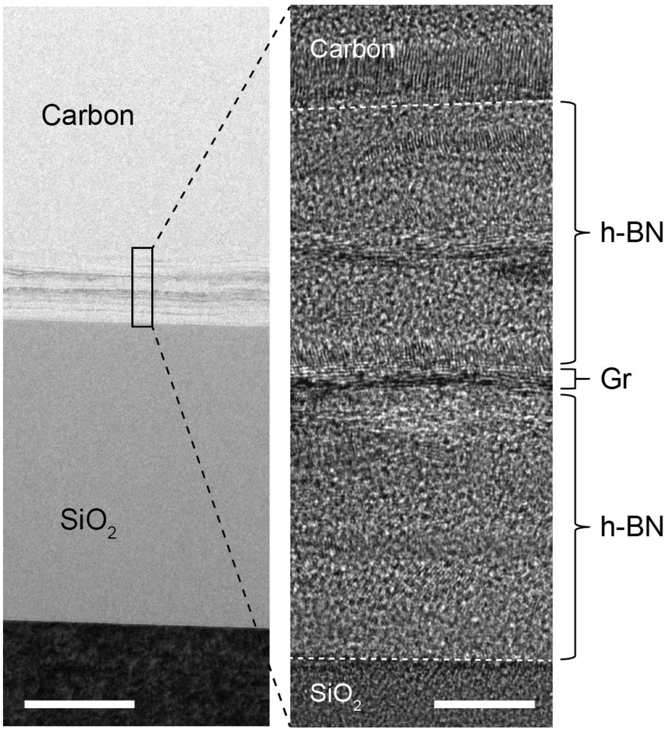

Supplementary Fig. 2. TEM image. Cross-sectional TEM images showing the structure of h-BN/Gr/h-BN. The magnified image (right) shows that the top and bottom multilayers of h-BN and three layers of Gr have thicknesses of ~30 nm and ~3 nm, respectively. The carbon coating on top prevents artifacts and damage caused by the focused ion beam (FIB) procedure for sample preparation for TEM imaging. Scale bars, 100 nm (left) and 10 nm (right).

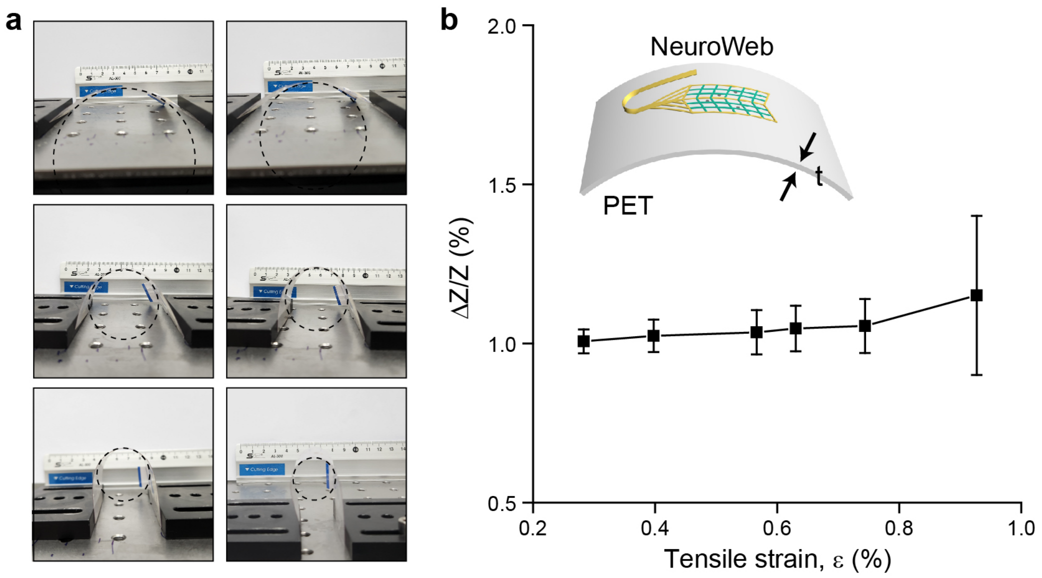

Supplementary Fig. 3. Mechanical reliability of NeuroWeb. a, A series of images illustrating the gradual increase in tensile strain experienced by NeuroWeb attached to the surface of a Polyethylene terephthalate (PET) substrate, as the curvature radius R is decreased from 35 to 10 mm. The PET substrate is bent with a thickness of t on a given radius R. The uniaxial tensile strain, ε, can be calculated using $\varepsilon = 100t / (2R + t)$. **b,** Normalized impedance change, $\Delta Z/Z$ (Z: initial impedance and ΔZ: changed impedance after the bending test), was measured as a function of tensile strain, ε. The impedance of NeuroWeb remained nearly unchanged up to a strain of 0.74%, while a few channels showed an increasing impedance at 0.92%. This result demonstrates the high mechanical reliability and stability of the device.

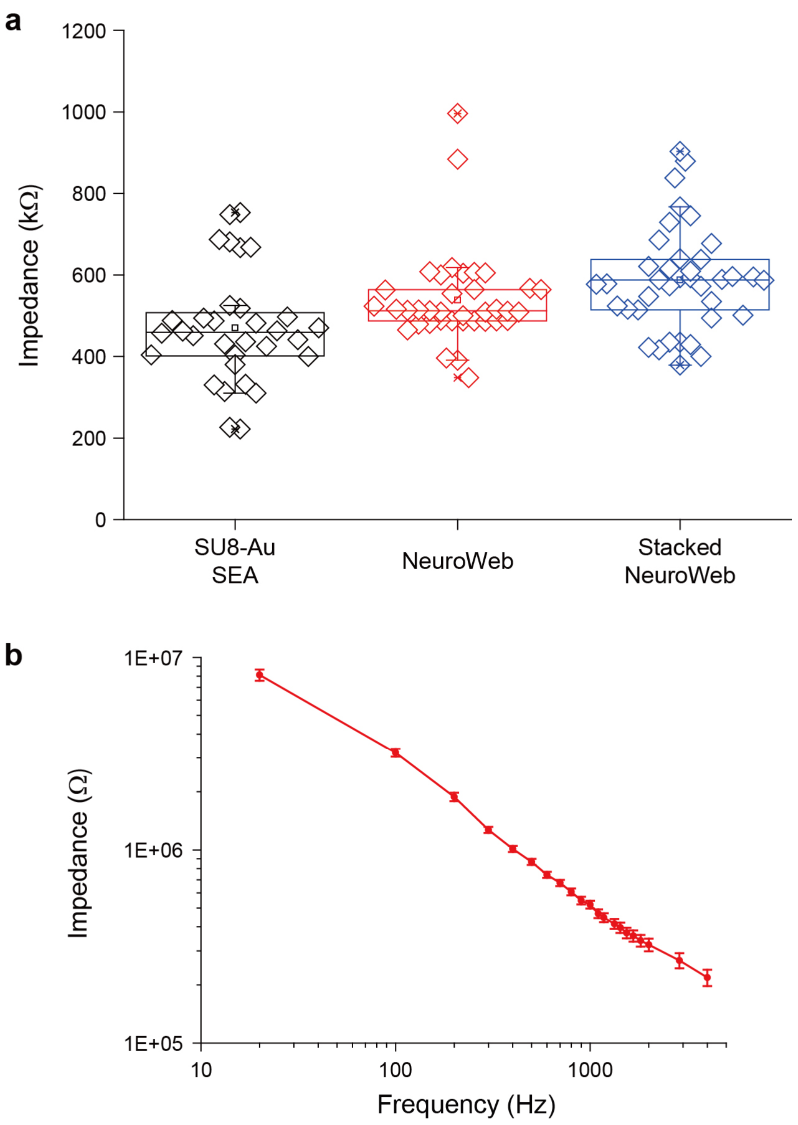

Supplementary Fig. 4. *In vitro* impedance measurement. a, Box-whisker plot of impedance values of SU8-Au SEA (black diamonds), original NeuroWeb (red diamonds) and stacked NeuroWeb (blue diamonds), measured by the Intan system at 1 kHz. Boxplots show mean (squares), median (horizontal lines), quartiles (boxes, 25-75%), and range (whiskers, 1-99%). **b,** Frequency-dependent impedance of NeuroWeb, measured from 20 Hz to 4000 Hz (N = 32 channels). The impedance values are high at low frequencies and low at high frequencies.

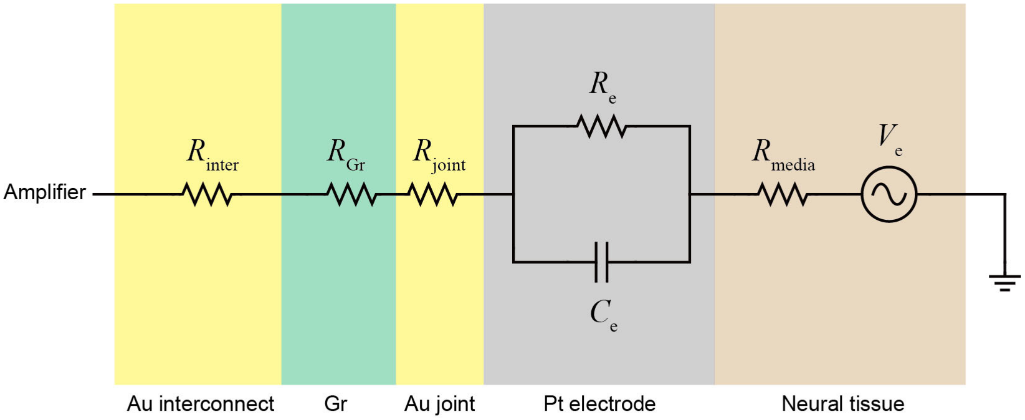

Supplementary Fig. 5. Modeling of the equivalent circuit. The NeuroWeb/tissue equivalent circuit assumes that neurons function like a voltage source (V_e). R_{media} denotes the resistance of the extracellular space. R_e and C_e indicate the leakage resistance and electrical double-layer capacitance of the interface between the Pt electrode and the tissue, respectively. R_{joint} is the resistance of the Au joint, and R_{Gr} is the resistance of the graphene line. R_{inter} is the resistance of the Au interconnect that connects to the amplifier.

h-BN has a large bandgap (5.97 eV) and a small lattice mismatch with graphene (1.7%). These properties of h-BN minimize surface charge traps between graphene and h-BN and enable high carrier mobility of graphene on h-BN. Therefore, NeuroWeb can record high signal-to-noise ratio (SNR) signals while being less affected by signal attenuation due to shank capacitance.

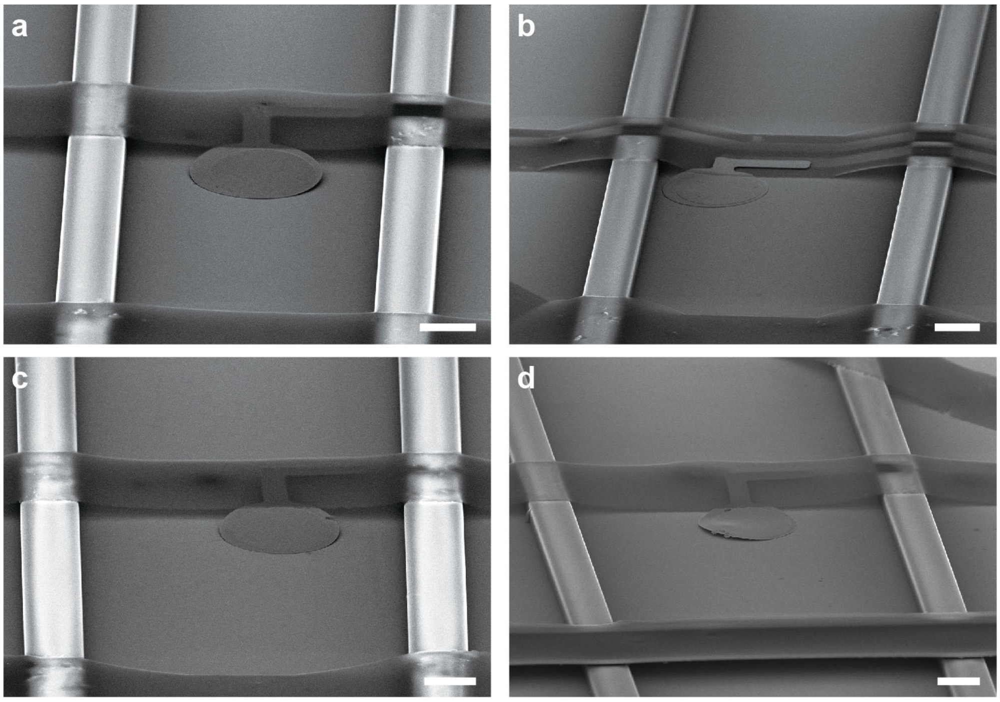

Supplementary Fig. 6. Flexibility experiment with NeuroWeb. SEM images of NeuroWeb transferred to periodic SU-8 lines with gap distances of 50 μm (**a**), 60 μm (**b**), 70 μm (**c**), and 80 μm (**d**) on the Au-coated substrate. All recording electrodes of NeuroWeb made contact with the Au-coated substrate. Scale bars, 10 μm.

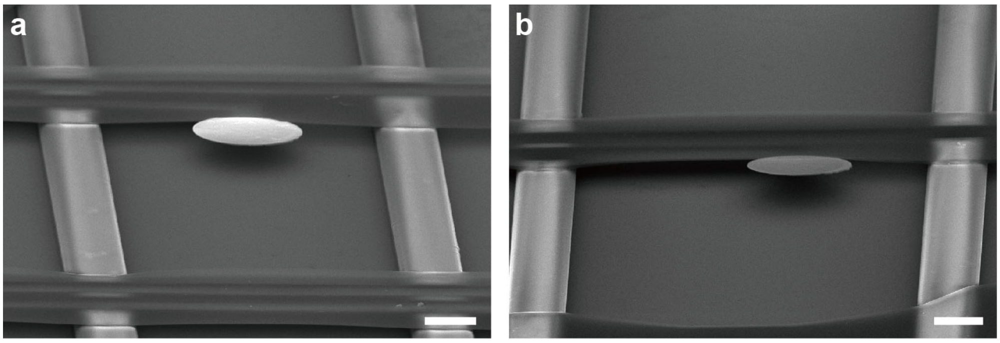

Supplementary Fig. 7. Flexibility experiment with SU8-Au SEA. SEM images of the SU8-Au SEAs transferred to periodic SU-8 lines with gap distances of 60 μm (**a**) and 80 μm (**b**) on the Au-coated substrate. The electrodes of the SU8-Au SEAs did not make contact with the Au-coated substrate. Scale bars, 10 μm.

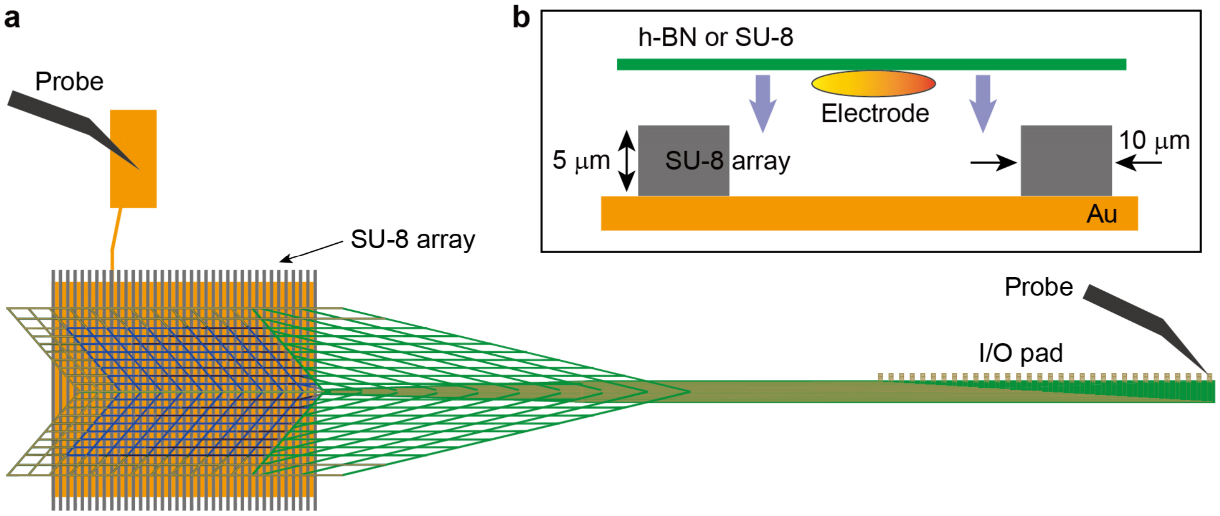

Supplementary Fig. 8. *In vitro* resistance measurement in the flexibility experiment. a, Top-view schematic of resistance measurement between the electrode and the Au-coated substrate (orange) with periodic SU-8 lines (gray). A voltage ranging from -1 V to 1 V is applied between the two probes for the I-V measurement. **b,** Side-view schematic showing a neural probe transferred to the Au-coated substrate with periodic SU-8 lines.

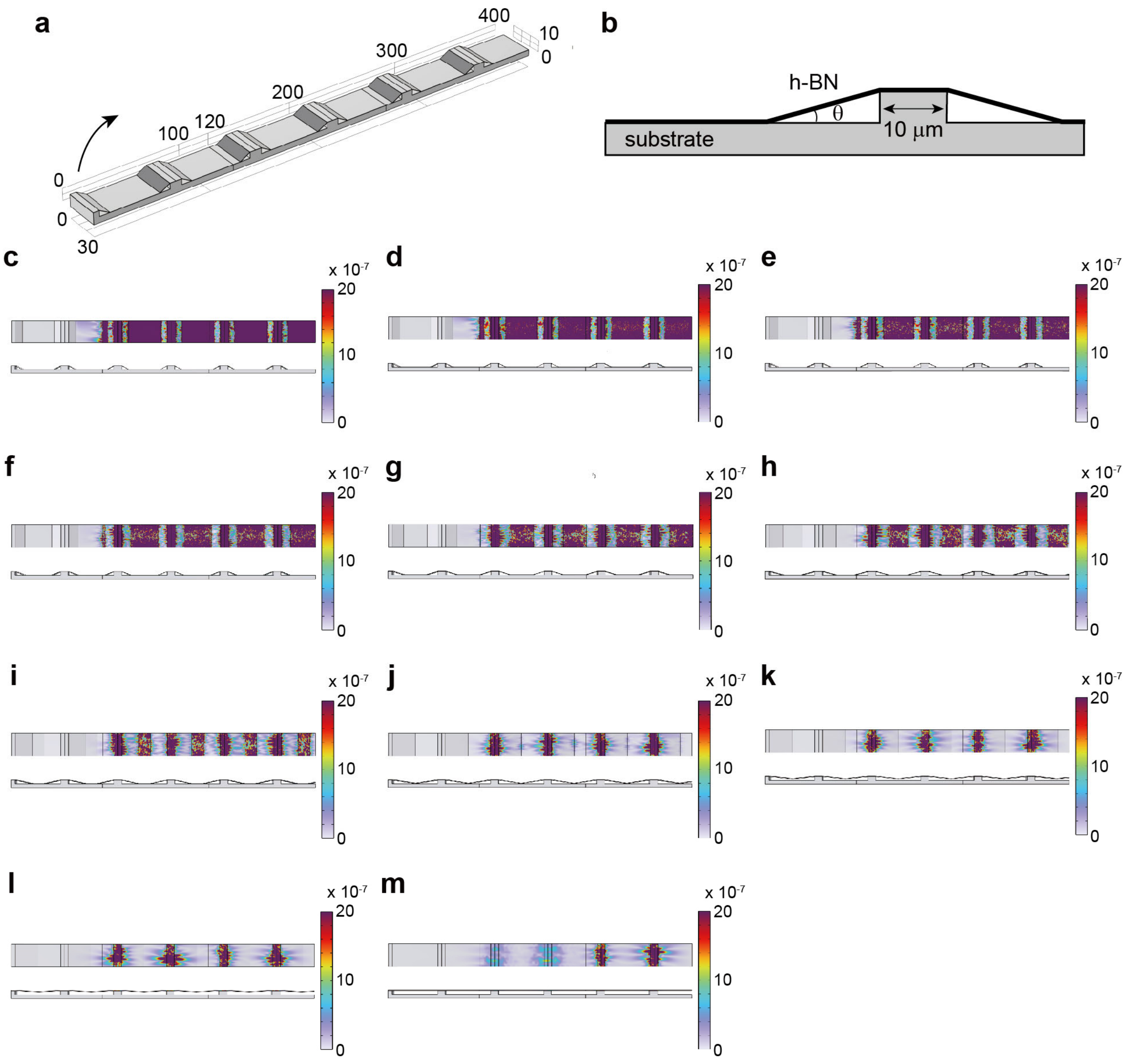

Supplementary Fig. 9. Numerical simulation of adhesive force with varying thickness of h-BN. a and b, Tilted-view (**a**) and side-view (**b**) of the structure used in FEM simulation. The simulation was performed using the h-BN film attached to a biomaterial substrate. Periodic patterns with 5-μm height, 10-μm width, and 60-μm periodicity were introduced on top of the substrate. Initially, the left 120 μm of the h-BN film was separated from the substrate. We then calculated the total adhesive elastic energy for the entire h-BN film when the remaining part of h-BN was completely separated from the substrate. The numbers in **a** are in μm. In addition, θ in **b** is the angle between the h-BN film and substrate before separation, which was identified using SEM images of the actually fabricated structures (Supplementary Fig. 6). **c-m**, Calculated top-

view and side-view von Mises stresses at (h-BN thickness, θ) of (**c**) (60 nm, 30.0°), (**d**) (120 nm, 28.1°), (**e**) (200 nm, 25.5°), (**f**) (300 nm, 22.3°), (**g**) (400 nm, 19.1°), (**h**) (500 nm, 16.0°), (**i**) (600 nm, 12.8°), (**j**) (700 nm, 9.6°), (**k**) (800 nm, 6.4°), (**l**) (900 nm, 3.2°), and (**m**) (1000 nm, 0°). The colored scale bars indicate adhesive force in N/m^2.

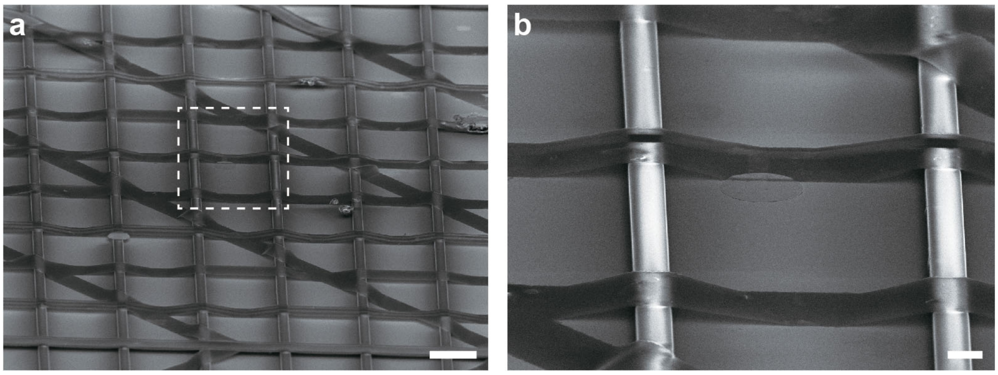

Supplementary Fig. 10. Flexibility experiment with stacked NeuroWeb. a, SEM image of the stacked NeuroWeb transferred to periodic SU-8 lines with an 80-μm gap distance on the Au-coated substrate. **b,** Magnified SEM image of the dashed white box in **a**. The electrodes of the stacked NeuroWeb made contact with the Au-coated substrate. Scale bars, 50 μm (**a**) and 10 μm (**b**), respectively.

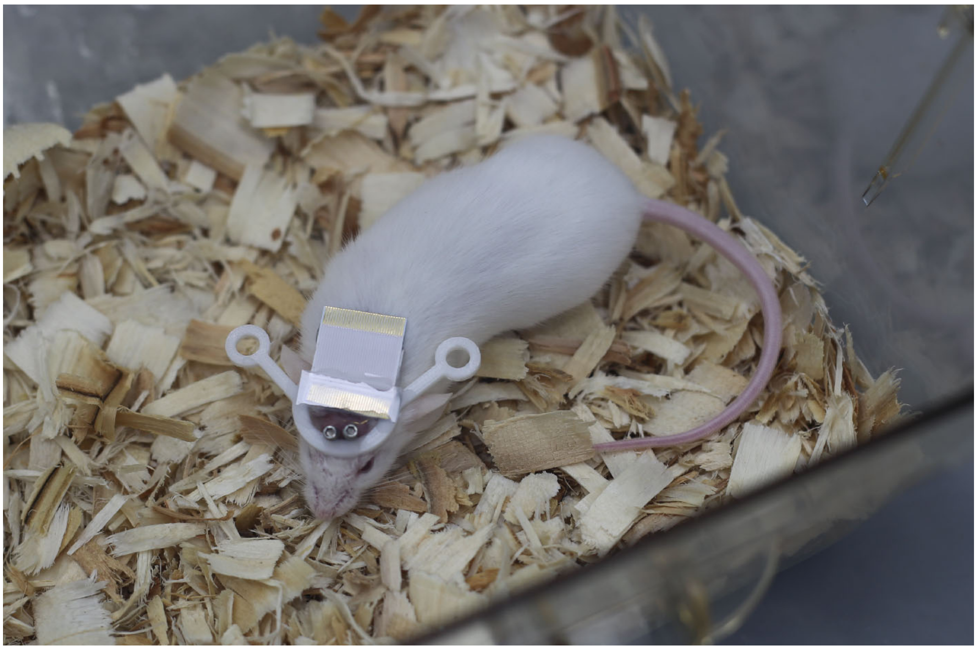

Supplementary Fig. 11. Photograph of a free-moving mouse with NeuroWeb. The active region of NeuroWeb was mounted on the mouse brain cortex, and the I/O pads were electrically connected to the metal pins of the FFC by using a direct-contact method. The total weight of NeuroWeb, head stage, and FFC was ~1.5 g. For recording, a PCB and a voltage amplifier will be connected to the FFC.

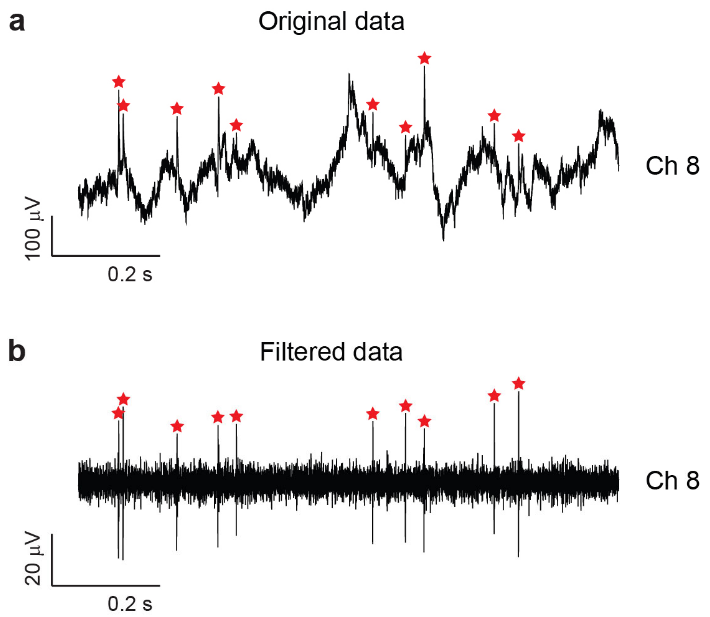

Supplementary Fig. 12. Original and filtered traces. We present the (**a**) original and (**b**) band-pass filtered (250−6000 Hz) trace data, to provide a more comprehensive view of the recordings and to enhance the reliability of the measured results. The red stars, which are exemplar upward deflections characteristic of detected extracellular spikes, agree well in the original and filtered traces.

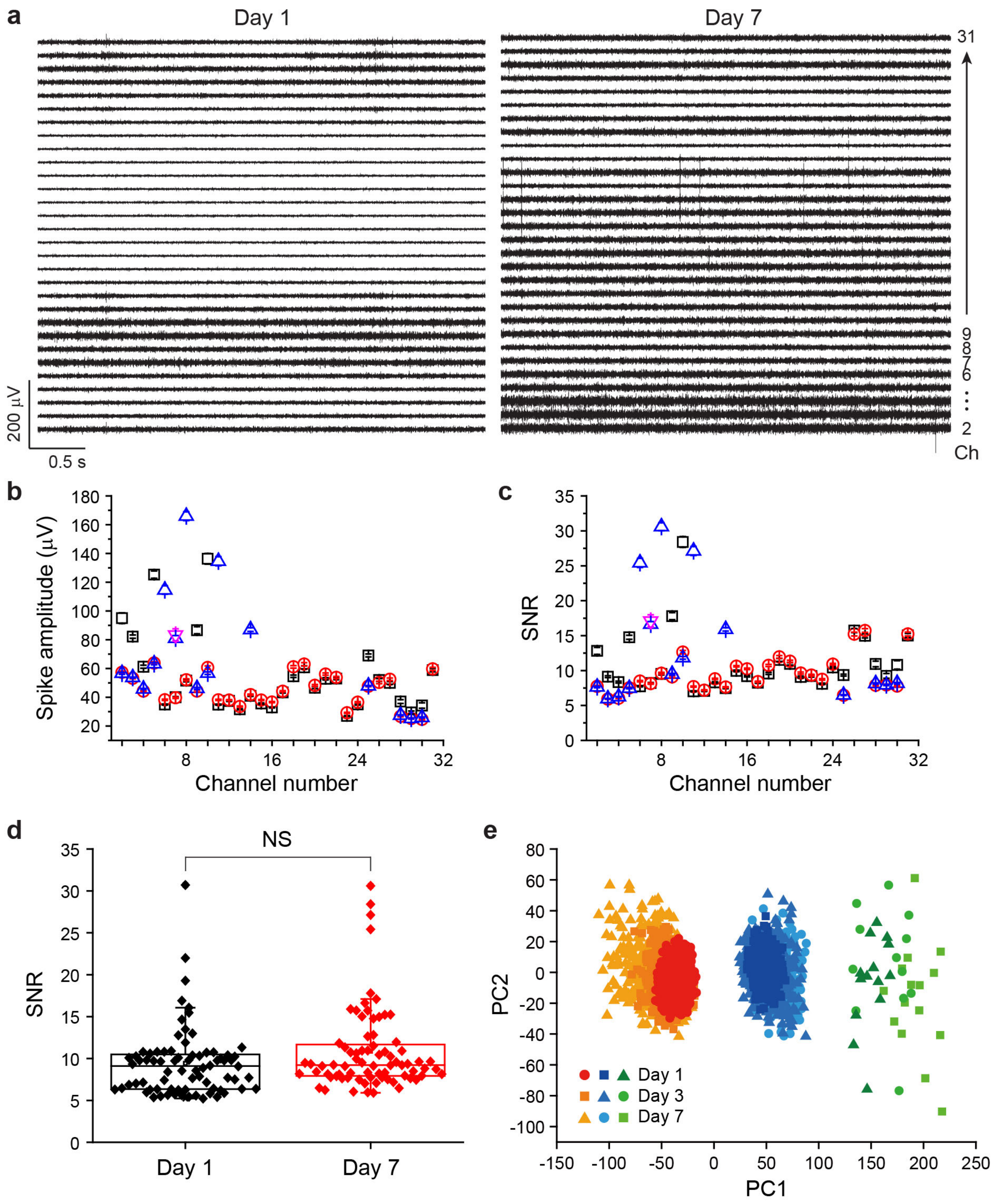

Supplementary Fig. 13. Analysis of recording data with NeuroWeb. a, Band-pass filtered (250−6000 Hz) extracellular spike recordings from the surface of the cortex at day 1 (left) and day 7 (right) after surgery. **b** and **c,** Spike amplitudes (**b**) and SNRs (**c**) of recorded single-unit action potentials for each channel at day 7 after surgery. Each distinct color corresponds to a neuron

uniquely identified for each channel. Error bars denote s.e.m. Data obtained at day 1 after surgery are shown in Figs. 3i and j. **d,** Boxplots with individual data points of SNRs obtained at day 1 and 7 after surgery, from 30-channel electrodes of NeuroWeb. NS: not significant ($P > 0.05$, two-sided t-test). Boxplots show mean (squares), median (horizontal lines), quartiles (boxes, 25-75%), and range (whiskers, 1-99%). **e,** Time evolution of representative single-unit spikes of Ch 8 in Fig. 3k, which was clustered by principal component analysis (PCA) over a week after surgery. The x- and y-axes denote the first and second principal components, respectively.

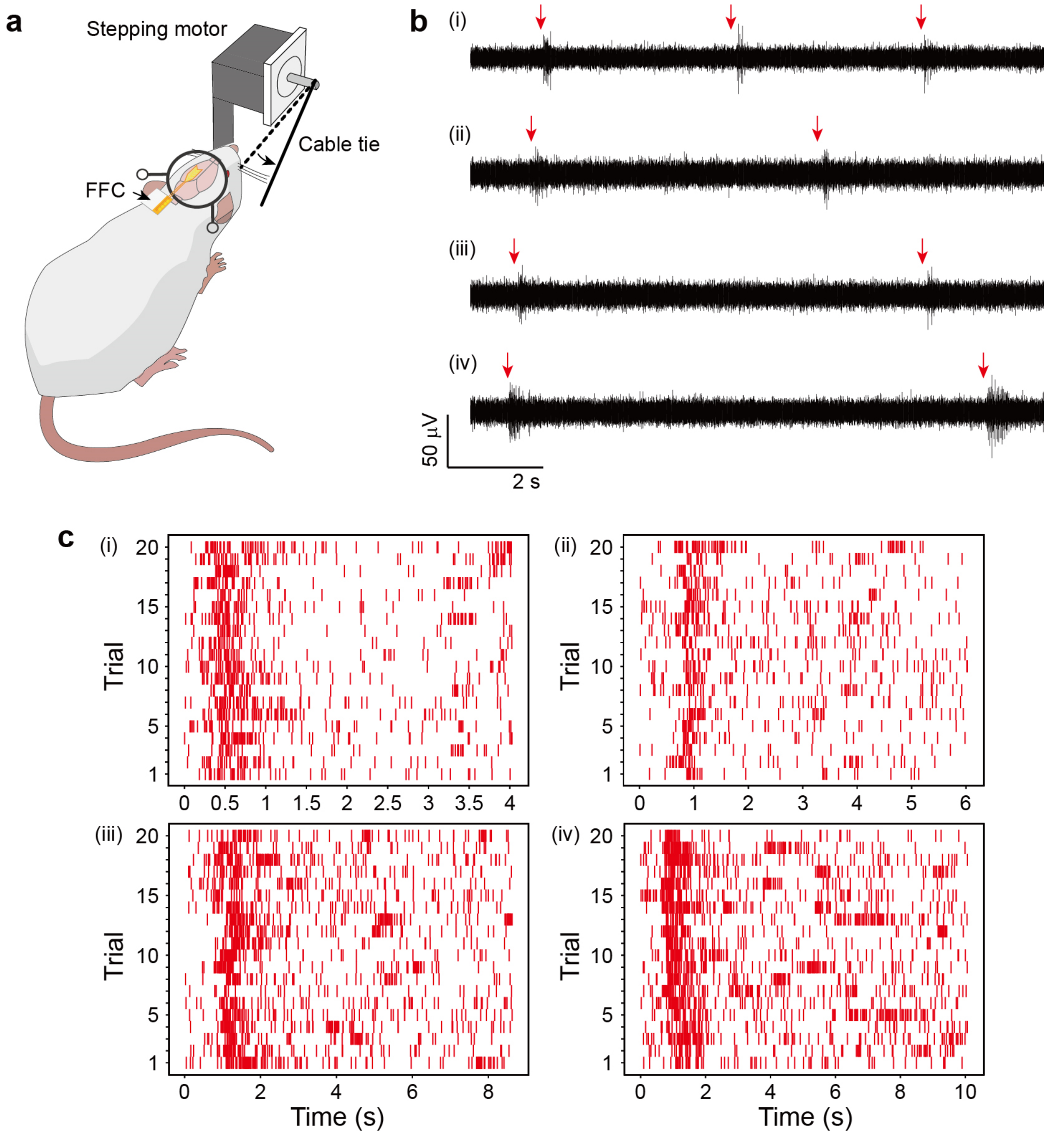

Supplementary Fig. 14. Whisker stimulation and electrophysiological recording. a, Schematic of whisker stimulation using a stepping motor and a cable tie. The cable tie was attached to the rotating shaft of the stepping motor, which was controlled by Arduino and rotated regularly. The mouse equipped with NeuroWeb was restrained with a fixed head stage and had its right whisker stimulated. **b,** Recorded spike trains with NeuroWeb during whisker stimulation at four intervals of (i) 4.04, (ii) 6.04, (iii) 8.63, and (iv) 10.07 seconds. The red arrows indicate the whisker-stimulation pulses. **c,** Spike raster plots as a function of time, for 20 stimulation trials at four intervals of (i) 4.04, (ii) 6.04, (iii) 8.63, and (iv) 10.07 seconds.

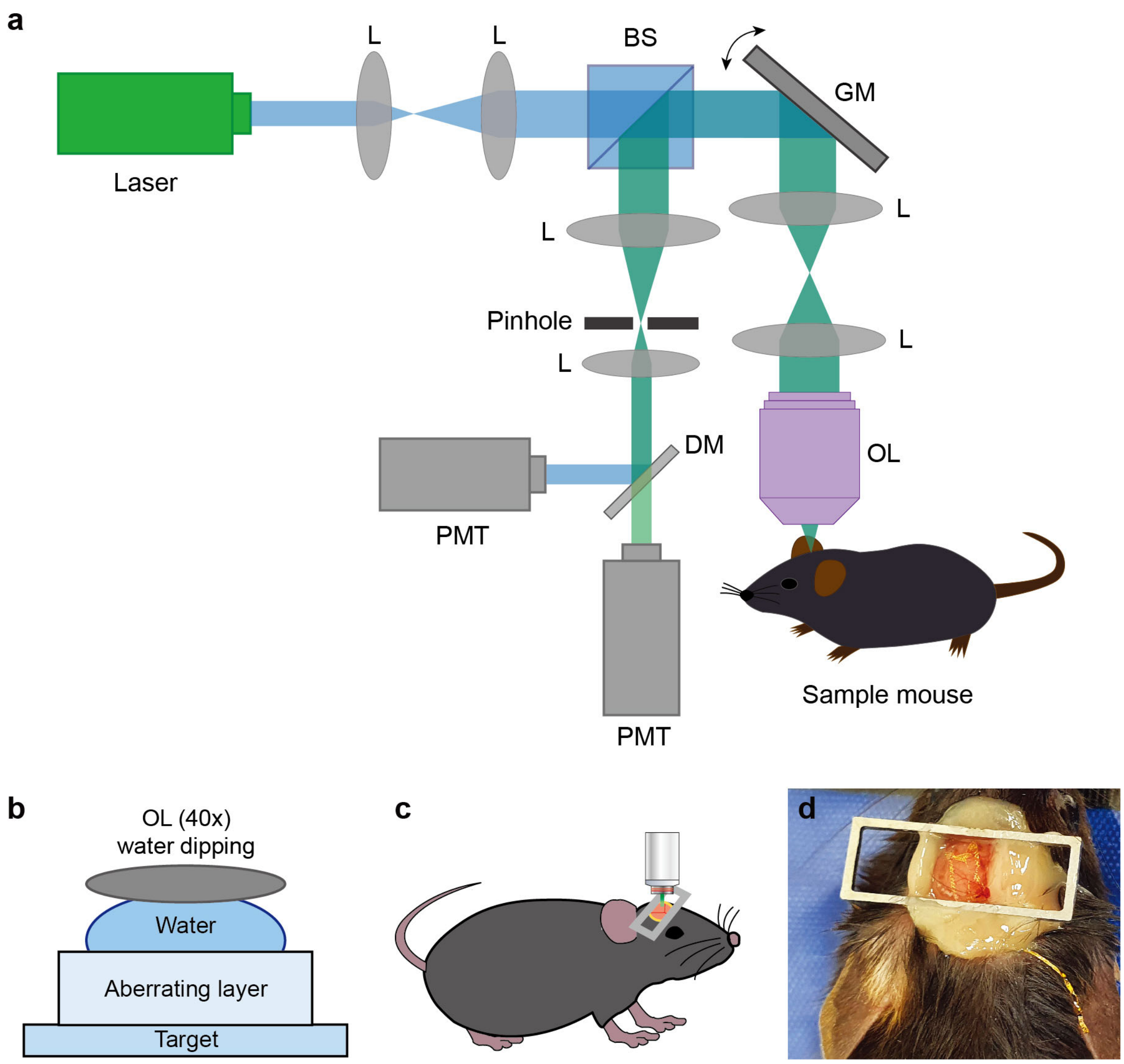

Supplementary Fig. 15. *In vivo* confocal fluorescence microscopy imaging. a, Schematic of the experimental setup for *in vivo* confocal fluorescence microscopy imaging[1]. L: lens, OL: objective lens, BS: beam splitter, GM: galvo mirror, DM: dichroic mirror, and PMT: photomultiplier tube. **b,** Layout of the sample geometry. **c,** Schematic of the sample mouse used for *in vivo* confocal fluorescence microscopy imaging. **d,** Photograph of the mouse framed with dental cement for *in vivo* confocal fluorescence microscopy imaging.

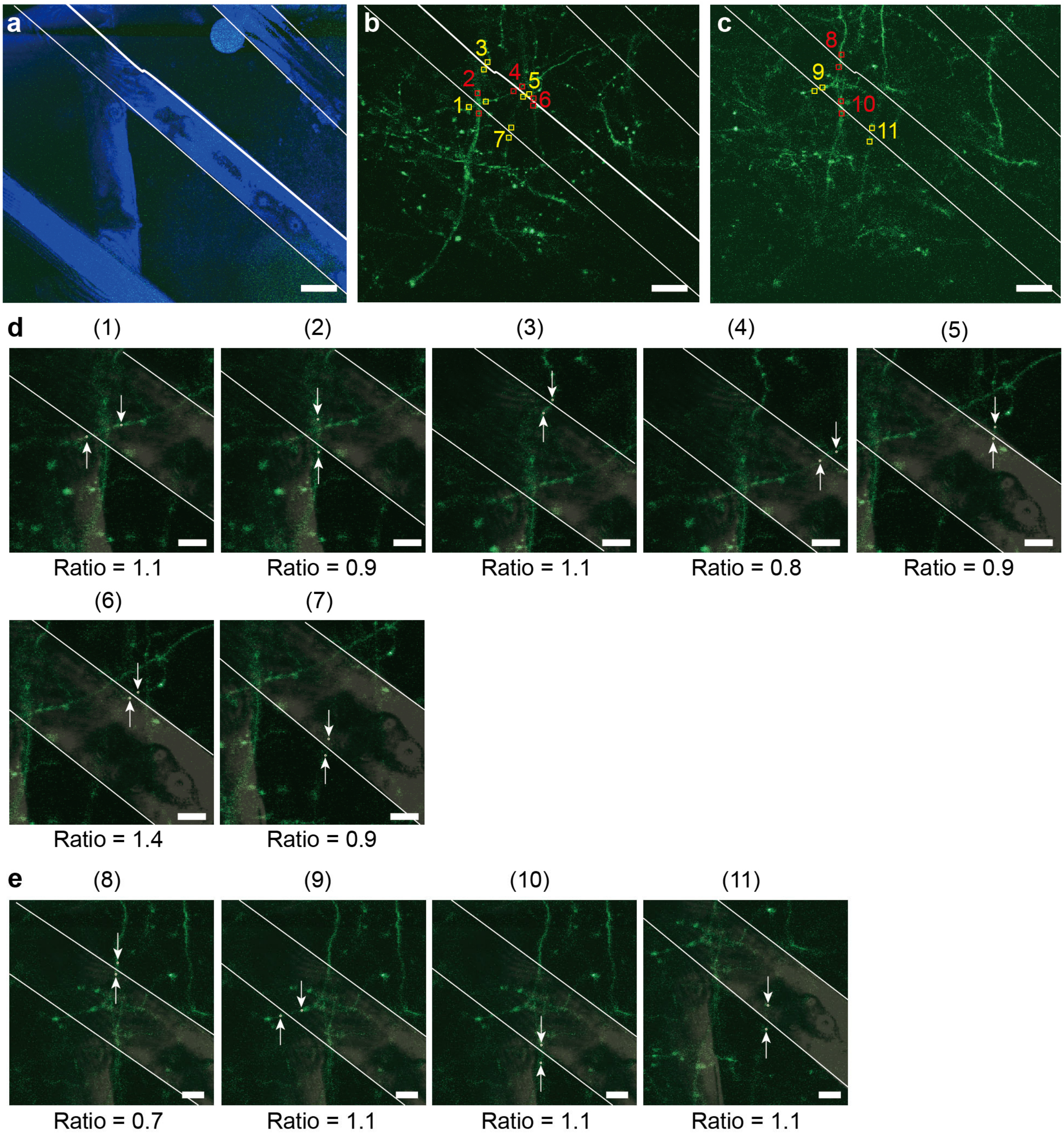

Supplementary Fig. 16. *In vivo* confocal fluorescence imaging with NeuroWeb. a, *In vivo* confocal image obtained at a depth of 19 μm. Scale bar, 20 μm. **b** and **c,** *In vivo* confocal fluorescence images obtained at depths of 94 μm (**b**) and 105 μm (**c**). Scale bars, 20 μm. **d,** Magnified images and corresponding energy ratios of (1) to (7) in **b**. Scale bars, 10 μm. **e,** Magnified images and corresponding energy ratios of (8) to (11) in **c**. Scale bars, 10 μm. The energy ratios were estimated by comparing the fluorescence intensity of a myelinated axon

underneath the BN-Gr ribbon with the intensity just outside the ribbon, which were indicated by two white arrows in each image.

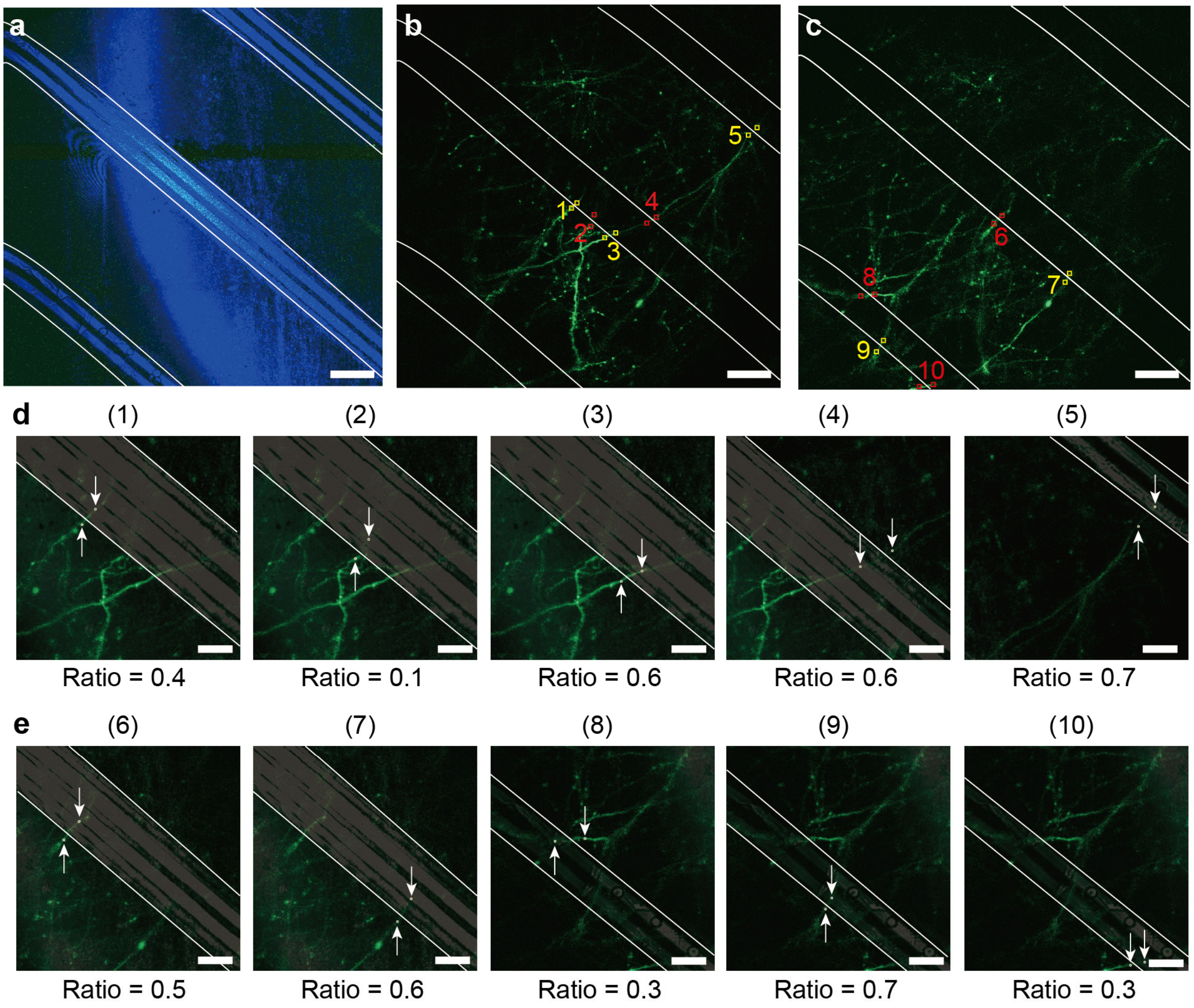

Supplementary Fig. 17. *In vivo* confocal fluorescence imaging with SU8-Au SEA. a, *In vivo* confocal image obtained at a depth of 9 μm. Scale bar, 20 μm. **b** and **c,** *In vivo* confocal fluorescence images obtained at depths of 47 μm (**b**) and 55 μm (**c**). Scale bars, 20 μm. **d,** Magnified images and corresponding energy ratios of (1) to (5) in **b**. Scale bars, 10 μm. **e,** Magnified images and corresponding energy ratios of (6) to (10) in **c**. Scale bars, 10 μm. The energy ratios were estimated by comparing the fluorescence intensity of a myelinated axon underneath the SU8-Au ribbon with the intensity just outside the ribbon, which were indicated by two white arrows in each image.

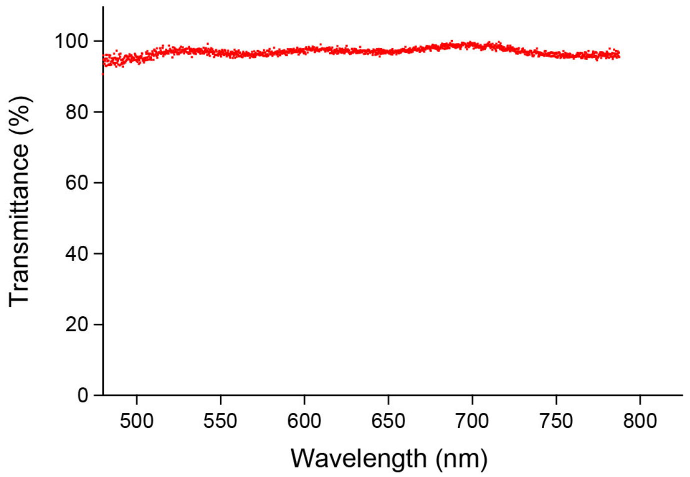

Supplementary Fig. 18. Measurement of transmittance. The transmittance of the h-BN/Gr/h-BN ribbon was measured over a wide visible wavelength range (480-780 nm). The measurement showed a uniform and high transmittance, with an average transmittance of 96% in this visible range.

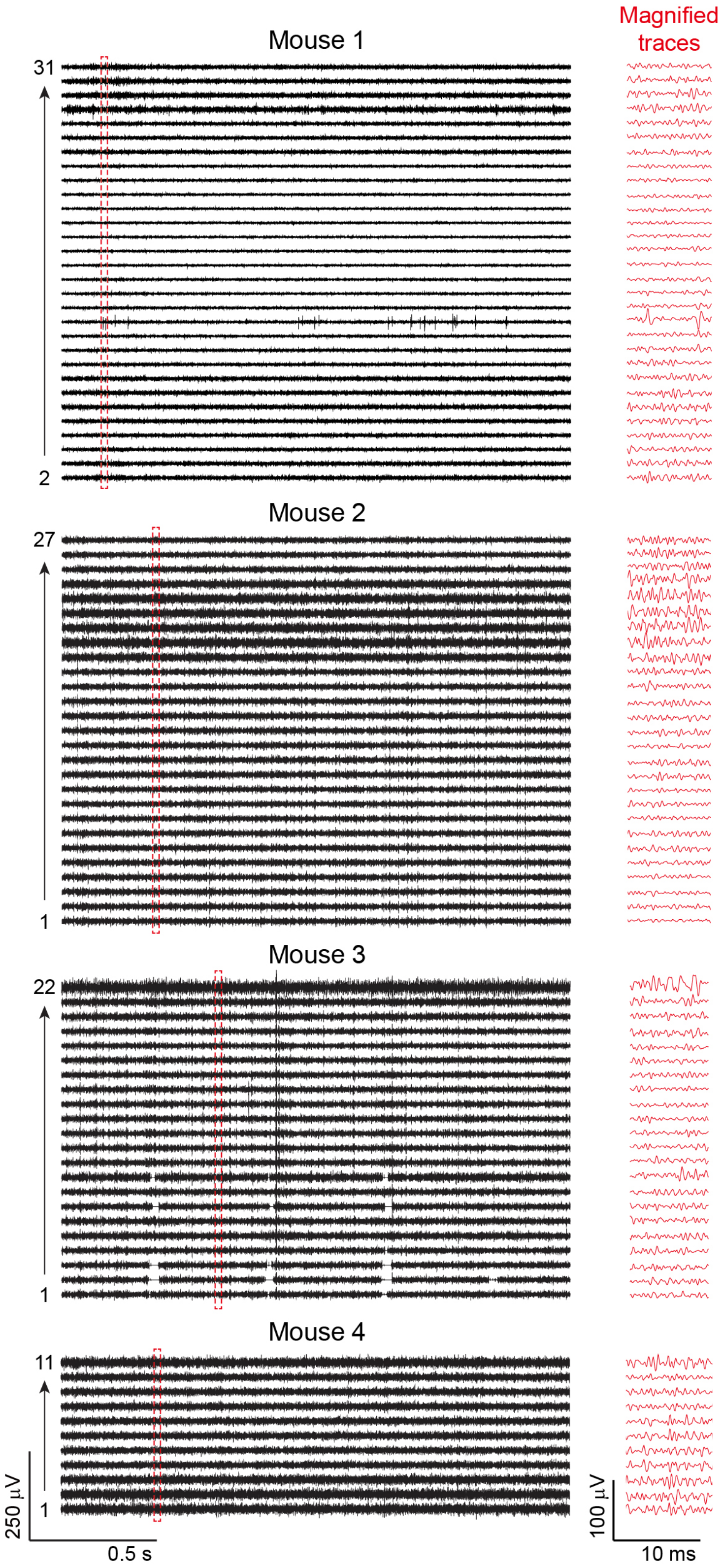
Magnified traces
Mouse 1
31
2
Mouse 2
27
1
Mouse 3
22
1
Mouse 4
11
1
250 µV
0.5 s
100 µV
10 ms

Supplementary Fig. 19. Extracellular recordings using NeuroWeb. Band-pass filtered (250−6000 Hz) extracellular spike recordings by NeuroWeb from the mouse cortex ($N = 4$) at day 3 after surgery. Data in Supplementary Fig. 13 were obtained from Mouse 1. We also display magnified views of the same spike action potentials for 10 ms (right red traces), which correspond to the red dashed boxes in the left traces. The activation time delays between channels are clearly visible, indicating that signals were recorded from distinct neurons.

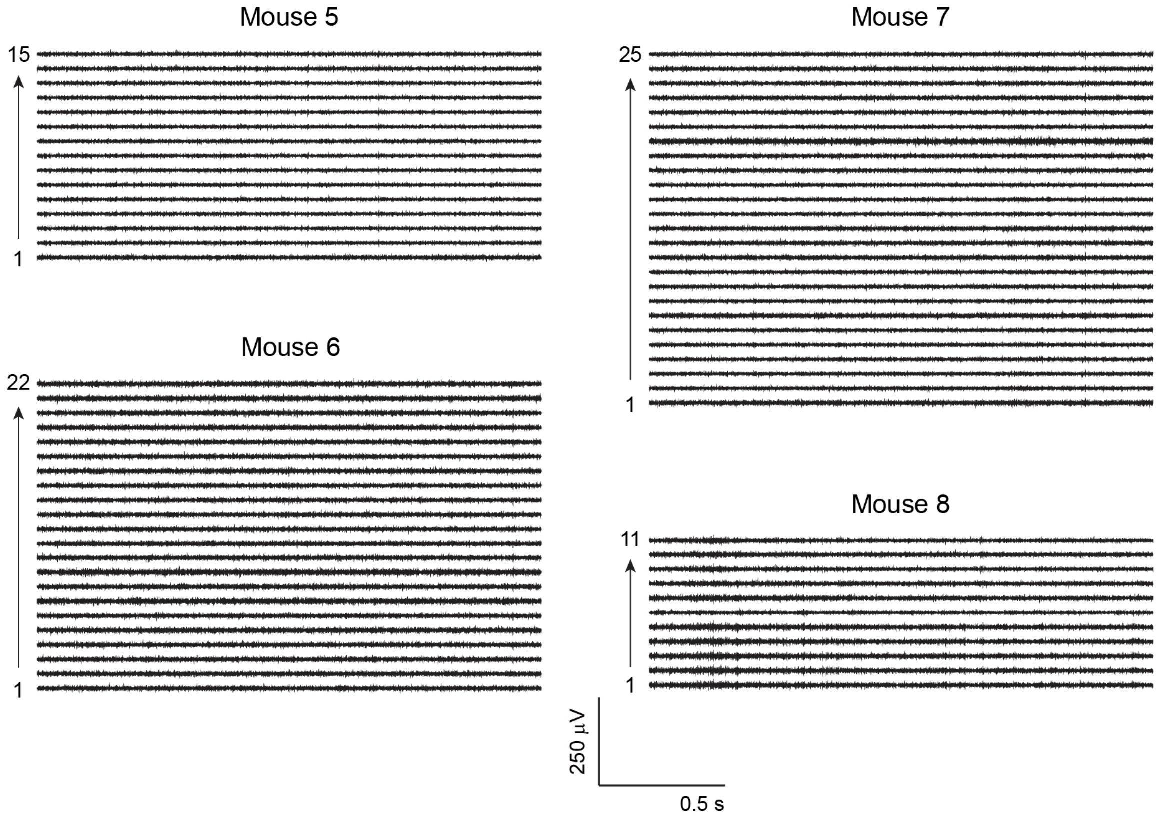

Supplementary Fig. 20. Extracellular recordings using SU8-Au SEA. Band-pass filtered (250−6000 Hz) extracellular spike recordings by SU8-Au SEA from the mouse cortex ($N = 4$) at day 3 after surgery.

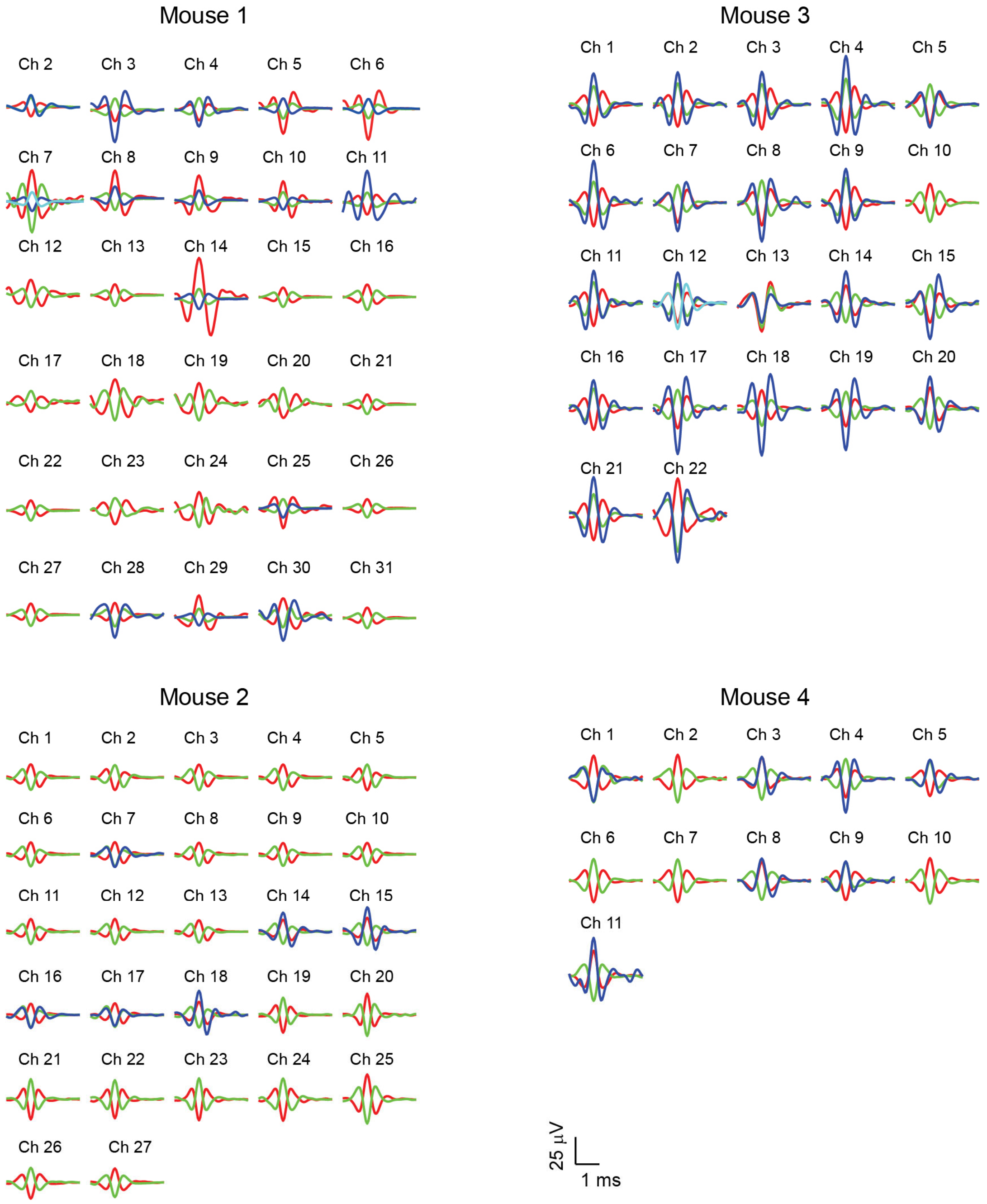

Supplementary Fig. 21. Overlay of sorted and clustered spikes in NeuroWeb. Clustered spikes from the data shown in Supplementary Fig. 19. We analyzed a total of 90 channels in four mice at day 3 after surgery.

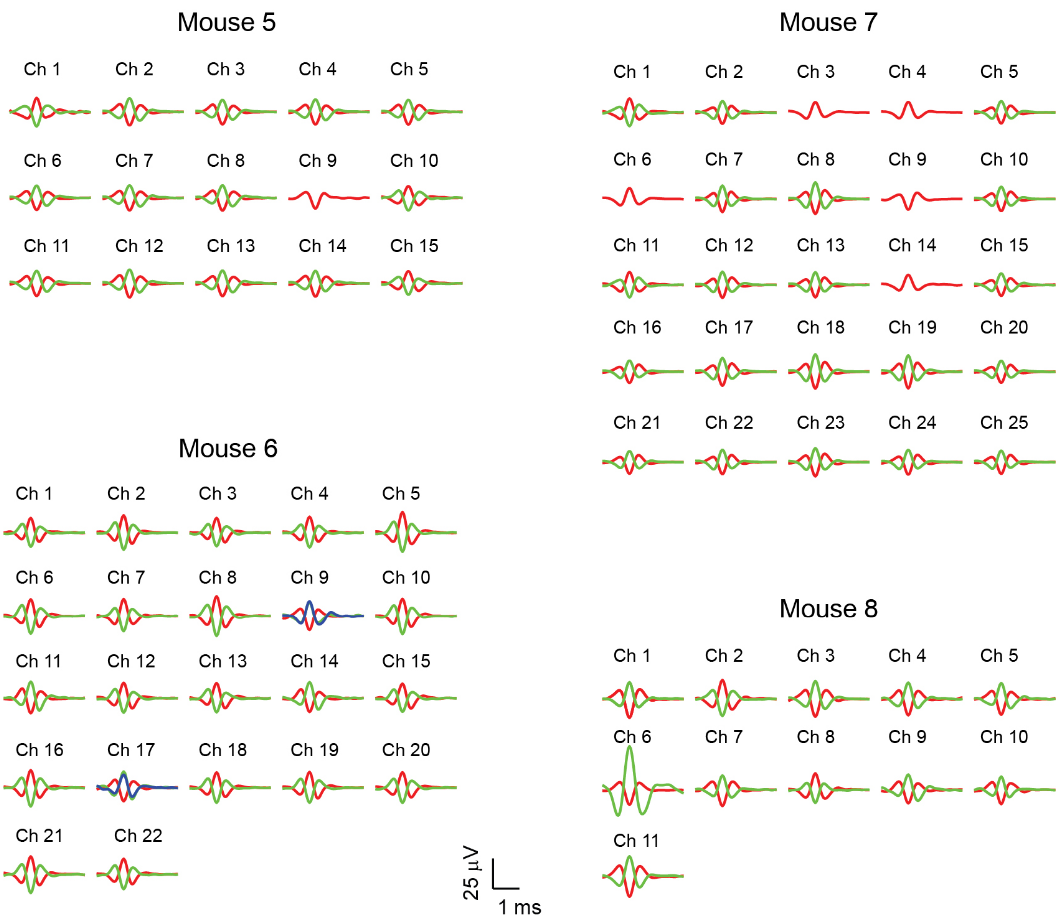

Supplementary Fig. 22. Overlay of sorted and clustered spikes in SU8-Au SEA. Clustered spikes from the data shown in Supplementary Fig. 20. We analyzed a total of 73 channels in four mice at day 3 after surgery.

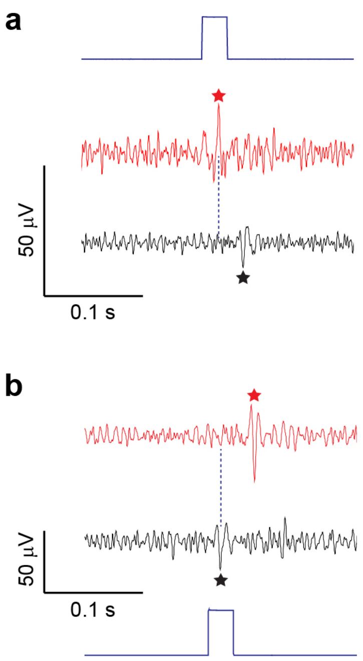

Supplementary Fig. 23. Magnified traces. a and **b,** Magnified band-pass filtered extracellular spike recordings of Figs. 5f (**a**) and 5g (**b**) from the S1 (NeuroWeb A, black traces) and the Cb (NeuroWeb B, red traces). A pulsed laser (blue line) optically stimulates the Cb (**a**) or S1 (**b**). The dotted lines are added to display the time difference between the evoked and responded spikes (★).

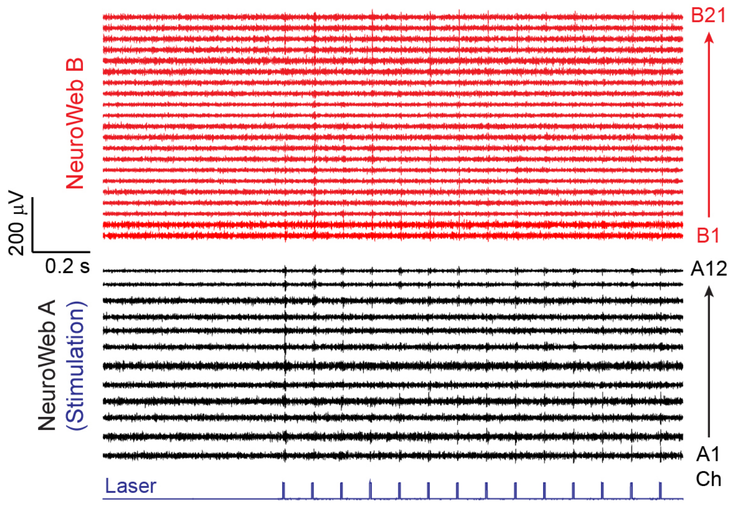

Supplementary Fig. 24. Extracellular recordings with optical stimulation. Band-pass filtered (250−6000 Hz) extracellular spike recordings from the surfaces of S1 (NeuroWeb A, black traces) and Cb (NeuroWeb B, red traces), when the S1 was optically stimulated.

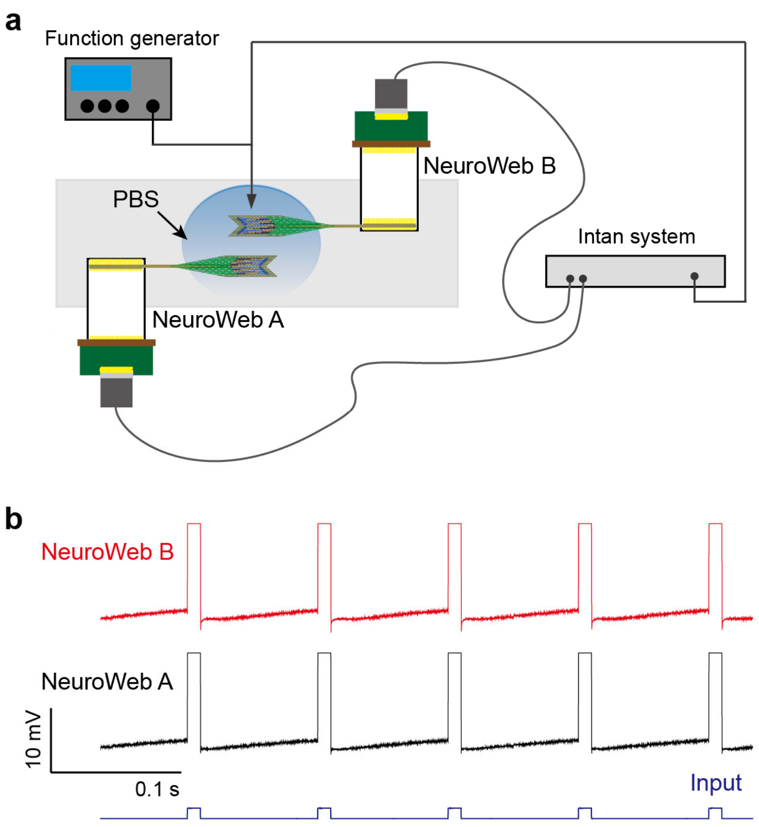

Supplementary Fig. 25. Electrical synchronization between two NeuroWebs. a, Schematic of *in vitro* measurement setup using two NeuroWebs. Two NeuroWebs on the slide glass were electrically connected to two individual FFCs using a direct-contact method. FFCs were also connected to the Intan recording system via custom-made printed circuit boards (PCB) and Intan 32-channel amplifiers. A function generator applied a voltage pulse with a frequency of 10 Hz and a pulse width of 5 ms (same condition as in Fig. 5) to NeuroWebs via phosphate buffered saline (PBS). The Intan recording system was synchronized with the function generator. **b,** Signals recorded from NeuroWebs A (black) and B (red), which are compared with the input voltage (blue). There is no latency between the signals measured in the two NeuroWebs.

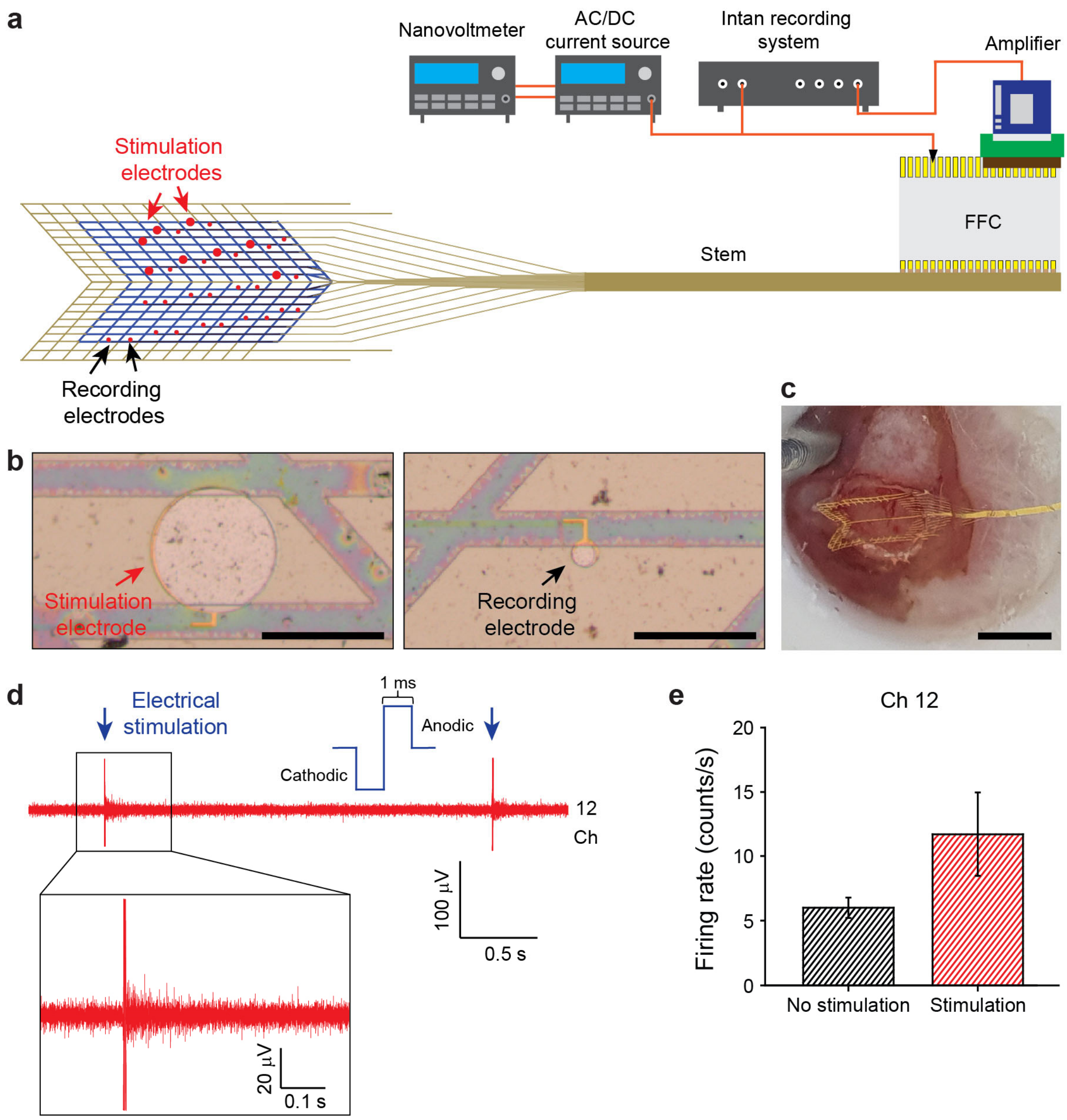

Supplementary Fig. 26. Electrical stimulation and recording using NeuroWeb. a, Schematic illustration of NeuroWeb for internal electrical stimulation and recording, consisting of 8 stimulation Pt electrodes with a radius of 50 μm and 24 recording Pt electrodes with a radius of 10 μm. I/O pads are connected to FFC for both stimulation and recording. Electrical pulses are applied via the stimulation Pt electrodes using an AC/DC current source and a nanovoltmeter. **b,** Optical microscope images of fabricated stimulation (left) and recording (right) Pt electrodes. Scale bars, 100 μm. **c,** Photograph of NeuroWeb placed on the S1. Scale bar, 3 mm. **d,** Representative trace (Ch 12) recorded following the application of electrical biphasic pulses (current 30 μA, charge

density 381 μC/cm^2, pulse width 1 ms, and frequency 0.4 Hz) via the stimulation Pt electrodes. A magnified trace (black box) demonstrates distinct neural activity in response to electrical stimulation. **e,** Comparison of the firing rates in Ch 12 before (black) and after (red) electrical stimulation.

	SNR
This work (NeuroWeb)	9.3
Fu, T. M. et al. [Ref. 2]	8.7
Jun, J. et al. [Ref. 3]	8.1
Kozai, T. et al. [Ref. 4]	8
Shin, H. et al. [Ref. 5]	5.3
Mohanty, A. et al. [Ref. 6]	4.73
Park, S. et al. [Ref. 7]	2.9

Supplementary Table 1. Comparison of SNRs in neural probes.

Supplementary References

1. Yoon, S., Lee, H., Hong, J. H., Lim, Y. S. & Choi, W. Laser scanning reflection-matrix microscopy for aberration-free imaging through intact mouse skull. *Nat. Commun.* **11**, 5721 (2020).

2. Fu, T. M., Hong, G., Viveros, R. D., Zhou, T. & Lieber, C. M. Highly scalable multichannel mesh electronics for stable chronic brain electrophysiology. *Proc. Natl. Acad. Sci. U. S. A.* **114**, E10046 (2017).

3. Jun, J. et al. Fully integrated silicon probes for high-density recording of neural activity. *Nature* **551**, 232–236 (2017).

4. Kozai, T. et al. Ultrasmall implantable composite microelectrodes with bioactive surfaces for chronic neural interfaces. *Nat. Mater.* **11**, 1065–1073 (2012).

5. Shin, H. et al. Multifunctional multi-shank neural probe for investigating and modulating long-range neural circuits in vivo. *Nat. Commun.* **10**, 3777 (2019).

6. Mohanty, A. et al. Reconfigurable nanophotonic silicon probes for sub-millisecond deep-brain optical stimulation. *Nat. Biomed. Eng.* **4**, 223–231 (2020).

7. Park, S. et al. Adaptive and multifunctional hydrogel hybrid probes for long-term sensing and modulation of neural activity. *Nat. Commun.* **12**, 3435 (2021).
