# Supplementary material for: The ultra-thin, minimally invasive surface electrode array NeuroWeb for probing neural activity
Source: Nat Commun. 2023 Nov 4;14:7088. doi: 10.1038/s41467-023-42860-9 (PMC10625630; doi:10.1038/s41467-023-42860-9)
Supplement: Supplementary file 3 — Reporting Summary [file 41467_2023_42860_MOESM3_ESM.pdf]

nature portfolio

Corresponding author(s): Hong-Gyu Park

Last updated by author(s): Oct 2, 2023

Reporting Summary

Nature Portfolio wishes to improve the reproducibility of the work that we publish. This form provides structure for consistency and transparency in reporting. For further information on Nature Portfolio policies, see our Editorial Policies and the Editorial Policy Checklist.

Statistics

For all statistical analyses, confirm that the following items are present in the figure legend, table legend, main text, or Methods section.

n/a	Confirmed	
☐	☒	The exact sample size (*n*) for each experimental group/condition, given as a discrete number and unit of measurement
☐	☒	A statement on whether measurements were taken from distinct samples or whether the same sample was measured repeatedly
☐	☒	The statistical test(s) used AND whether they are one- or two-sided<br>*Only common tests should be described solely by name; describe more complex techniques in the Methods section.*
☒	☐	A description of all covariates tested
☐	☒	A description of any assumptions or corrections, such as tests of normality and adjustment for multiple comparisons
☐	☒	A full description of the statistical parameters including central tendency (e.g. means) or other basic estimates (e.g. regression coefficient) AND variation (e.g. standard deviation) or associated estimates of uncertainty (e.g. confidence intervals)
☐	☒	For null hypothesis testing, the test statistic (e.g. F, t, r) with confidence intervals, effect sizes, degrees of freedom and P value noted<br>*Give P values as exact values whenever suitable.*
☒	☐	For Bayesian analysis, information on the choice of priors and Markov chain Monte Carlo settings
☒	☐	For hierarchical and complex designs, identification of the appropriate level for tests and full reporting of outcomes
☒	☐	Estimates of effect sizes (e.g. Cohen's *d*, Pearson's *r*), indicating how they were calculated

Our web collection on statistics for biologists contains articles on many of the points above.

Software and code

Policy information about availability of computer code

Data collection	IntanRHD2000 system was used to acquire electrophysiology data. No custom software or code was used for data collection.
Data analysis	WaveClus 2.0 and custom MATLAB code were used for electrophysiology data analysis.

For manuscripts utilizing custom algorithms or software that are central to the research but not yet described in published literature, software must be made available to editors and reviewers. We strongly encourage code deposition in a community repository (e.g. GitHub). See the Nature Portfolio guidelines for submitting code & software for further information.

Data

Policy information about availability of data

All manuscripts must include a data availability statement. This statement should provide the following information, where applicable:

- Accession codes, unique identifiers, or web links for publicly available datasets
- A description of any restrictions on data availability
- For clinical datasets or third party data, please ensure that the statement adheres to our policy

All the data supporting the findings of this study are available within this Article and its Supplementary Information. Any additional information can be obtained from the corresponding author on reasonable request. Source data are provided with this paper.

Research involving human participants, their data, or biological material

Policy information about studies with human participants or human data. See also policy information about sex, gender (identity/presentation), and sexual orientation and race, ethnicity and racism.

Reporting on sex and gender	This is not relevant to our study.
Reporting on race, ethnicity, or other socially relevant groupings	This is not relevant to our study.
Population characteristics	This is not relevant to our study.
Recruitment	This is not relevant to our study.
Ethics oversight	This is not relevant to our study.

Note that full information on the approval of the study protocol must also be provided in the manuscript.

Field-specific reporting

Please select the one below that is the best fit for your research. If you are not sure, read the appropriate sections before making your selection.

☒ Life sciences ☐ Behavioural & social sciences ☐ Ecological, evolutionary & environmental sciences

For a reference copy of the document with all sections, see nature.com/documents/nr-reporting-summary-flat.pdf

Life sciences study design

All studies must disclose on these points even when the disclosure is negative.

Sample size	The sample size for each experiment is detailed in the Methods section (Fabrication of NeuroWeb and Vertebrate animal subjects).
Data exclusions	No data were excluded from the analysis.
Replication	We provided the n number for each experiment in the main text.
Randomization	Mice were randomized into different experimental groups.
Blinding	This is not relevant to our study. Our experiment has nothing to do with blinding.

Reporting for specific materials, systems and methods

We require information from authors about some types of materials, experimental systems and methods used in many studies. Here, indicate whether each material, system or method listed is relevant to your study. If you are not sure if a list item applies to your research, read the appropriate section before selecting a response.

Materials & experimental systems

n/a	Involved in the study	
☒	☐	Antibodies
☒	☐	Eukaryotic cell lines
☒	☐	Palaeontology and archaeology
☐	☒	Animals and other organisms
☒	☐	Clinical data
☒	☐	Dual use research of concern
☒	☐	Plants

Methods

n/a	Involved in the study	
☒	☐	ChIP-seq
☒	☐	Flow cytometry
☒	☐	MRI-based neuroimaging

Animals and other research organisms

Policy information about studies involving animals; ARRIVE guidelines recommended for reporting animal research, and Sex and Gender in Research

Laboratory animals	Adult (8-week) female CD-1 mice (Charles River Laboratories, Wilmington, MA) were used as vertebrate animal subjects in this study

Laboratory animals	for brain surface electrophysiology and whiskers stimulation. The Thy1-EGFP line M (Jackson Labs #007788) mice (3-month) were used as vertebrate animal subjects for in vivo confocal fluorescence imaging. Adult (8-week) male Thy1-ChR2-YFP mice (Jackson Laboratory) were used as vertebrate animal subjects for the optical stimulation of genetically modified neurons and electrophysiological recording.
Wild animals	No wild animals were used.
Reporting on sex	Female and male mice were used.
Field-collected samples	The study did not involve samples collected from field.
Ethics oversight	All animal experiments were approved by the Korea University Institutional Animal Care & Use Committee (KUIACUC2019-0024).

Note that full information on the approval of the study protocol must also be provided in the manuscript.
